# Gray matter volume microstructural alterations in chronic post-burn pruritus: a voxel-based morphometry analysis using 3D T1-weighted imaging

**DOI:** 10.3389/fnins.2025.1629878

**Published:** 2025-09-04

**Authors:** Zhi-kai Lu, Xiao-yan Li, Yin Huang, Wan-li Guo, Pei-yi Bai, Lu Liu, Jin-feng Zhu, Rui Wang

**Affiliations:** ^1^Department of CT Room, General Hospital of Tisco, The Sixth Hospital of Shanxi Medical University, Taiyuan, Shanxi, China; ^2^Shanghai Research Institute for Intelligent Autonomous Systems, Tongji University, Shanghai, China; ^3^Department of Burns, General Hospital of Tisco, The Sixth Hospital of Shanxi Medical University, Taiyuan, Shanxi, China; ^4^Clinical Discipline Construction Center, Shanxi Medical University, Taiyuan, Shanxi, China; ^5^Department of Medical Imaging, Shanxi Medical University, Taiyuan, Shanxi, China

**Keywords:** chronic post-burn pruritus, voxel-based morphometry analysis, 3D T1-weighted imaging, microstructural changes, gray matter volume

## Abstract

**Background:**

Chronic post-burn pruritus (CPBP) refers to a highly prevalent as well as debilitating problem after burn injuries, substantially impairing patients’ quality of life. Nevertheless, the precise pathological mechanisms underlying CPBP remain elusive. The present study seeks to investigate microstructural changes in gray matter among individuals with CPBP and to evaluate associations between these changes and clinical scale scores via voxel-based morphometry (VBM).

**Materials and methods:**

We recruited 20 people having CPBP and 20 healthy controls (HCs) who were of the same sex and age. T1-weighted imaging in three dimensions (3D-T1WI) was performed on each participant. Using VBM, volumes of gray matter in each region were measured. Volume differences between groups were measured, and it was methodically examined how the volume in particular brain regions correlated with clinical markers.

**Results:**

We found no discernible age difference between the groups (*P* > 0.05). Compared with HCs, patients with CPBP showed reduced gray matter volume across multiple brain regions (*P* < 0.05). These regions encompassed the bilateral parahippocampal gyri, right medial frontal gyrus, right middle frontal gyrus, left cerebellum posterior lobe, left fusiform gyrus, left superior occipital gyrus, left middle temporal gyrus, left cuneus, inferior parietal lobule, right medial frontal gyrus, and left superior temporal gyrus. Furthermore, correlation analysis showed a negative relationship between the individuals’ Self-Rating Depression Scale scores and the gray matter volume in the right superior temporal gyrus in patients with CPBP (*r* = −0.632, *P* = 0.003).

**Conclusion:**

The frontal, temporal, parietal, occipital, and cerebellar regions all exhibit a marked reduction in volume in response to CPBP.

## 1 Introduction

Throughout the world, burn injuries are among the most frequent traumatic injuries. In the course of and following wound healing, chronic or recurrent pruritus is frequently reported. When pruritus persists for more than 6 weeks after injury, it is defined as chronic post-burn pruritus (CPBP) (Ständer, 2016). Up to 96% of burn survivors experience three or more episodes of pruritus daily, with 94% considering their pruritus as intolerable (Parnell et al., 2012; Carrougher et al., 2013). The quality of life is negatively impacted by CPBP. However, the precise pathological mechanism underlying CPBP remains unclear, and effective treatment approaches are lacking (Andrade et al., 2024).

Neuroplasticity refers to its ability to structurally or functionally adapt to the external environment, experience, or injury. Post-burn pruritus is initiated by skin injury; however, neural plasticity may sustain pruritus in the late stages of burn (Goutos, 2013; Chung et al., 2020). It has been reported that there are changes in central nervous plasticity in burn survivors (Whife et al., 2021). Patients with chronic pruritus have been shown to have functional and microstructural changes in their brains, according to an increasing amount of neuroimaging data (Papoiu et al., 2014; Wang et al., 2018a,^2021^; Najafi et al., 2020). According to functional magnetic resonance imaging (MRI) studies, patients with CPBP exhibit reduced activity in both postcentral and precentral gyri but raised activity in the precuneus, medial superior frontal gyrus, middle frontal gyrus, left insula, and other areas (Lu et al., 2025). However, brain microstructural changes in patients with CPBP have not yet been explored, and to date, no research has specifically addressed MRI-based changes in gray matter for this population. Therefore, the investigation of CPBP patients using structural MRI enhances our understanding of the mechanisms underlying brain structural reorganization in such patients and may offer valuable insights for central nervous system-targeted interventions aimed at alleviating clinical symptoms, including post-burn pruritus.

High-resolution three-dimensional T1-weighted imaging (3D-T1WI) is an MRI-based technique that facilitates obtaining thin, continuous T1-weighted images in three dimensions. It distinctly visualizes the brain anatomy. Voxel-based morphometry (VBM) quantitatively calculates the gray matter volume at each Voxel in 3D-T1WI images and detects fine microstructural changes that are not visible on conventional MRI (Nemoto, 2017). VBM is commonly used to detect brain microstructure and has been widely applied to investigate gray matter changes in various diseases, including pruritus (Papoiu et al., 2014; Yang et al., 2020; Rechberger et al., 2022). In this study, both 3D-T1WI and VBM were utilized to investigate the microstructural gray matter changes among patients with CPBP. Based on the current structural and functional MRI findings related to chronic pruritus diseases, we hypothesize that patients with CPBP exhibit plasticity changes in the brain’s gray matter. These changes may correlate with clinical characteristics, including pruritus and the psychological status of CPBP patients.

## 2 Materials and methods

### 2.1 Participants

We included 49 prospectively recruited individuals, including 26 men diagnosed with CPBP who were admitted to the Department of Burns, General Hospital of Tisco, Taiyuan, Shanxi Province, China and 23 healthy controls (HCs) who were matched for age and sex and selected from the hospital’s physical examination center during the same period. This hospital’s Institutional Ethics Committee (Approval No. k202118) approved this protocol, and each participant signed a consent form.

The inclusion criteria for the CPBP group were: men; age between 30 and 60 years (All patients who were actually recruited and met the diagnostic criteria fell within this interval); right-handedness; burn injury covering > 8% of body surface; duration since burn history ≥ 3 months; pruritus diagnosis after burn; pruritus intensity score ≥ 4 points on the standard Visual Analogue Scale (VAS); and pain intensity score ≤ 2 points on the standard VAS. The VAS scores range from 0 to 10, with higher scores indicating more intense pruritus or pain. The exclusion criteria for both groups were: other chronic pruritic diseases; CNS disorders; neuropsychiatric disorders; and contraindications to MRI.

### 2.2 Research methods

Clinical data were collected from the CPBP group, including the duration of burn injury, percentage of burn area, pruritus intensity VAS score, pain intensity VAS score, Self-Rating Anxiety Scale (SAS) score (Zung, 1965, 1971), and Self-Rating Depression Scale (SDS) score. The SAS scores ranged from 25 to 100, with a score less than 50 considered normal. Higher scores indicate more severe anxiety symptoms. The SDS score ranged from 0.25 to 1.0, and a score lower than 0.5 was defined as no depression. Higher scores indicate more severe depressive symptoms.

### 2.3 MRI data acquisition

Using a 24-channel phased-array head coil, a GE 3.0 Tesla MRI machine (Discovery MR750w) was used for neuroimaging. The participants were asked to close their eyes and maintain a motionless head as well as body prior to scanning. A conventional cranial MRI was conducted to exclude intracranial pathology. With the following parameters, 3D-T1WI structural images were gathered using the brain volume imaging sequence: inversion time = 450 ms, time of repetition = 8.5 ms, time of echo = 3.2 ms, field of view = 256 mm × 256 mm, flip angle = 12°, slice thickness = 1 mm, voxel size = 3 mm × 3 mm × 3 mm, matrix size = 256 × 256, and interslice gap = 0 mm. Overall, 192 layers were scanned.

### 2.4 Image preprocessing and VBM analysis

The Computational Anatomy Toolbox 12 (CAT12)^1^ processed structural 3D-T1WI images within SPM12 software. Preprocessing involved:

(1)    Slice timing correction: Each subject’s T1W image was corrected for slice timing using the middle slice as the reference.
(2)    Motion correction: Data with head motion exceeding 2.0 mm of translation and/or 2.0° of rotation were excluded.
(3)    Spatial normalization: All data were normalized to a standard three-dimensional space using the EPI template in Montreal Neurological Institute (MNI) space.
(4)    A standard segmentation model separated all images into brain fluid, white matter, and gray matter.
(5)    The gray matter segments were normalized spatially to the Montreal Neurological Institute’s standard space.
(6)    The density map of gray matter was multiplied by the non-linear deformation parameter during spatial registration to obtain the modulated volumetric map.
(7)    A Gaussian kernel smoothed modulated images in the standard space (full width at half maximum = 6 × 6 × 6 in mm).

### 2.5 Statistical analysis

Software for data assessment was SPSS 25.0 and SPM12. Age and other continuous variables are shown by the mean ± standard deviation or median (interquartile range). *P* < 0.05 indicates statistical significance, and independent sample *t*-tests or Mann–Whitney U tests were used for comparing between groups. To take inter-individual variations into consideration, the total intracranial volume of each participant was employed as a covariate. The Gaussian Random Field theory corrected multiple comparisons, which has thresholds of *P* < 0.005 at the voxel level and *P* < 0.05 at the cluster level. Brain areas with significant volumetric differences served as regions of interest, and the correlation between their gray matter volume and clinical scale scores was analyzed. Considering the small sample size and non-normal distribution of most clinical variables, Spearman’s rank correlation ensured the robustness of results. It was deemed statistically significant when *P*-values were < 0.05.

## 3 Results

### 3.1 Differences in general clinical characteristics

After excluding participants based on MRI image quality and intracranial organic lesions, the data of 40 participants were included in the finale analyses. They comprised 20 HCs and 20 CPBP patients. We found no discernible age difference between the groups (*P* > 0.05) (Table 1). In the CPBP group, the average percentage of body surface area impacted by burn injuries was 46.7 ± 28.2%. Approximately 11.5 months was the mean disease duration (interquartile range [IQR]: 6.3–31.8 months). The median pruritus VAS score was 5.5 (IQR: 4.0–7.0), and pain VAS score was 1.0 (IQR: 0.0–6.0). The mean SAS score was 34.9 ± 9.4, and the SDS score was 0.4 (IQR: 0.3–0.5).

**TABLE 1:** Demographic and clinical characteristics of all participants.

Clinical characteristics	CPBP (*n* = 20)	HCs (*n* = 20)	*P*-value
Age (years)	42.3 ± 8.0	41.3 ± 5.1	0.92
Sex (m/f)	20/0	20/0	
Burn area (percentage of body surface area)	46.7% ± 28.2%	–	
Time since injury (months)	11.5 (6.3, 31.8)	–	
VAS pruritus score	5.5 (4.0, 7.0)	–	
VAS pain score	1.0 (0.0, 6.0)	–	
SAS score	34.9 ± 9.4	–	
SDS score	0.40 (0.3, 0.5)	–	

VAS, Visual Analogue Scale; SAS, Self-Rating Anxiety Scale; SDS, Self-Rating Depression Scale.

### 3.2 Comparison of VBM indexes between groups

**FIGURE 1:**
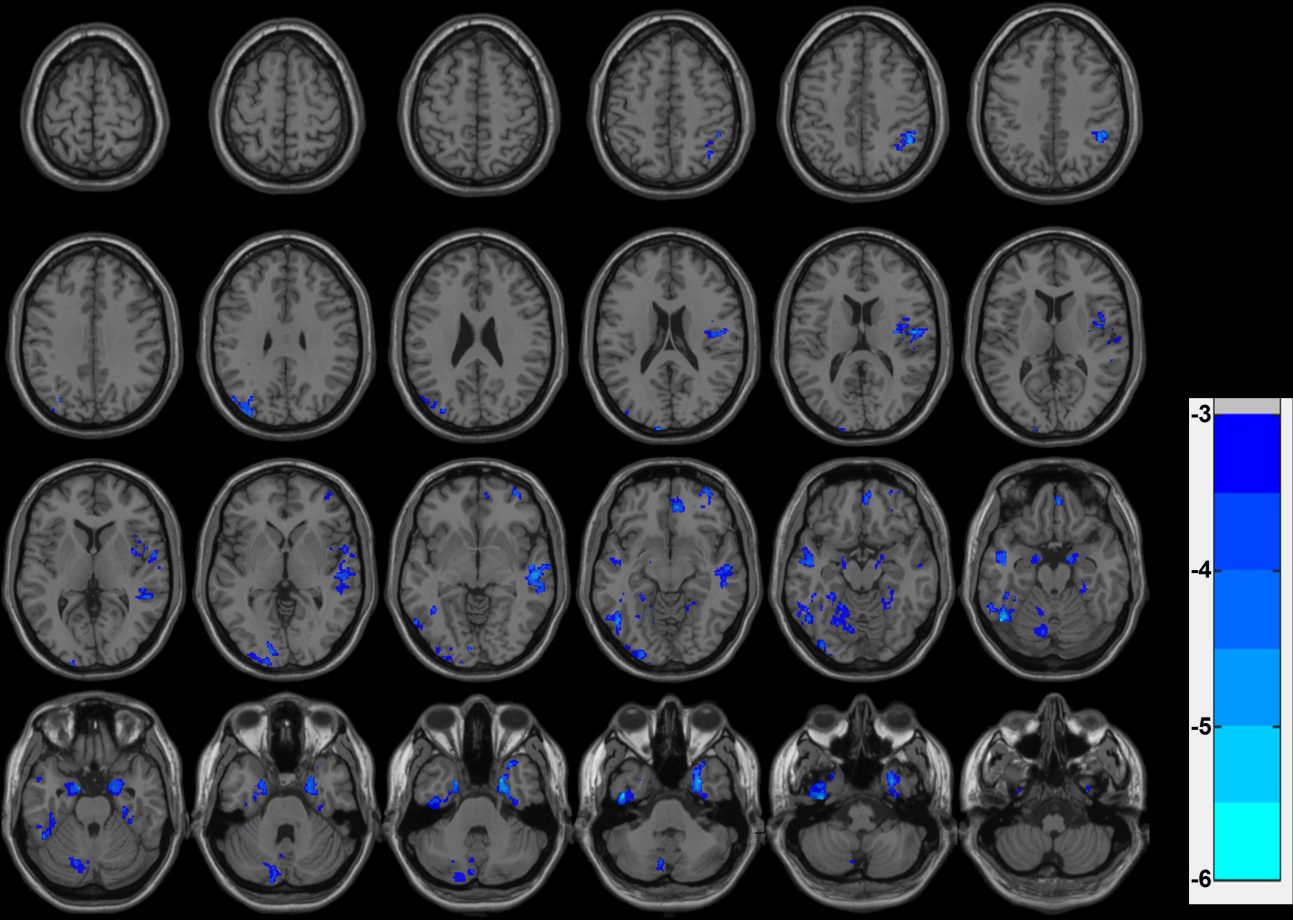
Results of statistical differences in gray matter VBM volume between the CPBP and HCs. Blue indicates brain regions exhibiting reduced gray matter volume, and the color bars correspond to *T*-values. Clump level GRF correction, *P* < 0.05.

The volume in several brain areas was considerably reduced in the CPBP group. These included the left fusiform gyrus, bilateral parahippocampal gyrus, left posterior cerebellar lobe, left middle temporal gyrus, right medial frontal gyrus, right middle frontal gyrus, left superior temporal gyrus, left cuneus, inferior parietal lobule, and left superior occipital gyrus (Figure 1 and Table 2).

**TABLE 2:** Results of statistically significant differences in gray matter volume between groups in the CPBP and HCs.

Brain region	Cluster size (voxels)	Peak MNI coordinate			Peak t-score
		x	y	z	
Left parahippocampal gyrus	1547	−45	−10.5	−40.5	−5.787
Right parahippocampal gyrus	2236	22.5	4.5	−36	−6.081
Left cerebellum posterior lobe	1553	−7.5	−69	−37.5	−4.113
Left fusiform gyrus	1285	−45	−10.5	−18	−5.773
Left middle temporal gyrus	512	51	0	−36	−4.383
Right medial frontal gyrus	423	6	42	−12	−4.591
Left cuneus	792	−10.5	−100.5	19.5	−4.470
Right middle frontal gyrus	342	36	55.5	−3	−4.606
Right superior temporal gyrus	2309	48	−10.5	16.5	−4.970
Left superior occipital gyrus	423	−39	−84	30	−4.552
Inferior parietal lobule	512	45	−48	43.5	−5.779

### 3.3 Correlation between VBM indexes and clinical indexes

**FIGURE 2:**
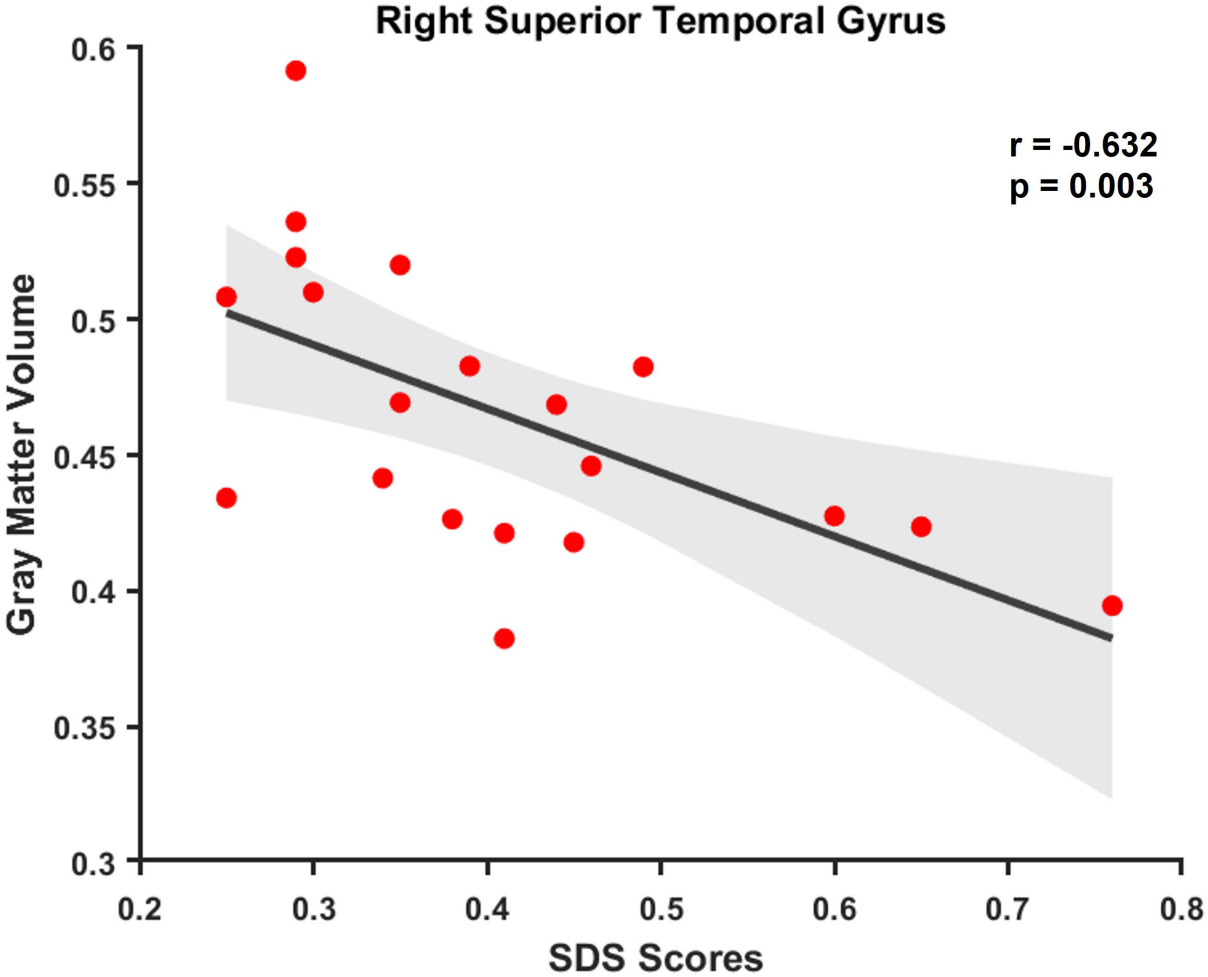
Correlation between gray matter volume in the right superior temporal gyrus and SDS scores for depression in patients with CPBP.

Figure 2 shows a negative link between the SDS scores and volume in the right superior temporal gyrus in people having CPBP (*r* = −0.632, *P* = 0.003) (Figure 2). No significant correlations were observed between clinical indexes, including burn areas, duration after burn injury, VAS scores for pruritic and pain, and SAS scores for anxiety, and gray matter volume in identified brain regions (*P* > *0.*05).

## 4 Discussion

This research is the first that we are aware of that uses 3D-T1WI via VBM to investigate the structural plasticity changes of gray matter in patients with CPBP. Studies have found that numerous brain regions showed markedly decreased volumes of gray matter in people having CPBP. In addition, some brain regions with gray matter volume differences are significantly correlated with depression scale scores in CPBP patients.

### 4.1 Reduced gray matter volume in the temporal lobe

The gray matter volume of the right superior temporal gyrus, left middle temporal gyrus and bilateral parahippocampal gyrus was significantly lower in the CPBP group than in the HCs. Interestingly, SDS scores were inversely connected with low volume in the right superior temporal gyrus, which is indicative of depression symptoms in CPBP patients. The hippocampus’s ability to function in relation to memory, emotion, and cognition is largely dependent on the parahippocampal gyrus (Jiang et al., 2021). Similarly, processing sensory information, controlling emotions, and social cognition are linked to the superior and middle temporal gyri (Gallagher and Frith, 2003). Reduced gray matter volumes in these brain regions may impair emotional processing and memory functions (Zola-Morgan et al., 1989). According to several studies the hippocampus and temporal lobe’s gray matter volume is often reduced in people having depression (Schmaal et al., 2016; Wise et al., 2017; Gray et al., 2020). Paolini et al. (2023) demonstrated that a suboptimal response to antidepressant was linked to reduced volume within the parahippocampal gyrus, middle temporal and right superior gyri, left hippocampus. These findings closely align with previous results on major depressive disorder (Schmaal et al., 2016; Wise et al., 2017; Gray et al., 2020). Furthermore, the mental health of burn survivors has been associated with the severity of scar pruritus, indicating that more severe pruritus is associated with greater psychological distress (Van Loey and Van Son, 2003). This study consisted of people having pruritus (moderate to severe). Additionally, severe pruritus exacerbates psychological issues. Consequently, it was hypothesized that patients with CPBP experiencing prolonged negative affect and other psychological challenges may exhibit structural plasticity changes within the CNS involved in processing itch-related affective information. Psychological status can modulate the symptoms of scar pruritus, and antidepressants alleviate chronic pruritus (Ständer et al., 2009). Hence, therapeutic strategies with the ability to structurally change brain regions should be targeted to manage the symptoms of patients with CPBP.

### 4.2 Low gray matter volume in the frontal lobe

Comparing CPBP patients to HCs, the volume in the right medial frontal gyrus was noticeably smaller. The medial frontal gyrus constitutes the superior frontal gyrus, which is central to motor planning and execution. Additionally, it regulates both motor and cognitive control (Rushworth et al., 2004; Nachev et al., 2008). The middle frontal gyrus has been implicated in maintaining attention, working memory, and self-control (Kjaer et al., 2002; Andrews et al., 2011). Prior research on pruritus has frequently reported abnormal activity within prefrontal regions (Dong and Dong, 2018; Roberts et al., 2019; Chen and Sun, 2020). Prefrontal cortex gray matter volume was also found to be decreased in one study examining pruritus associated with end-stage nephritis (Papoiu et al., 2014). The rewarding feeling of scratching during pruritus is mediated by rewards system activation, specifically involving the prefrontal cortex (Papoiu et al., 2013; Mochizuki et al., 2014; Vierow et al., 2015). Furthermore, patients with chronic pruritus exhibit substantially amplified addiction-related mechanisms, as evidenced by markedly increased activity in both motor and rewards-related brain regions during scratching, compared with HCs (Mochizuki et al., 2015). Burn survivors often experience burn-related sequelae, such as pruritus and a vicious cycle of itching and scratching. It is hypothesized that these symptoms may activate prefrontal regions involved in itching and scratching, potentially leading to microstructural plastic changes. The prefrontal cortex may serve as a non-invasive brain stimulation target for burn sequelae management (Thibaut et al., 2021). Our findings further support this hypothesis, highlighting the prefrontal cortex as a potential neural target for addressing post-burn pruritus.

### 4.3 Reduced gray matter volume in the occipital lobe

The left cuneus as well as left superior occipital gyrus, which are important processing centers for visual information, showed low volumes. The visual cortex functions abnormally (Wang et al., 2018b; Najafi et al., 2020; Dehghan Nayyeri et al., 2022) and has less gray matter volume (Papoiu et al., 2014) in people with chronic pruritus disease. The superior occipital gyrus contributes to the analysis and synthesis of visual stimuli (Roberts et al., 2019). The cuneus receives, processes, and transmits visual information from the primary visual cortex (Zhang et al., 2021; Geng et al., 2022). Responsiveness of the visual network cortex is highly dependent on somatosensory stimuli, and sensory disorders may alter visual cortex function (Chan et al., 2017). Compared to HCs patients with CPBP demonstrated heightened sensitivity to pruritus (van Laarhoven et al., 2016). Thus, it was hypothesized that abnormal pruritus perception in such patients may lead to structural neuroplasticity changes related to visual information processing.

### 4.4 Low gray matter volume in the parietal lobe

Both episodic memory and spatial cognition have been linked to the inferior parietal lobule, which comprise the angular and supramarginal gyri. Prior research has shown that both experimental and chronic pruritus are associated with aberrant inferior parietal lobule activation (Wang et al., 2018b; Roberts et al., 2019; Najafi et al., 2020). In people having psoriasis as well as mentally induced pruritus, a functional MRI study showed greater connectivity in the inferior parietal lobe than HCs (Najafi et al., 2020). A meta-analysis further suggested that the supramarginal gyrus shows significant differences between the brain activity in response to itch and pain stimuli, supporting that itch-specific somatosensory processing (Roberts et al., 2019). The present study demonstrates that the bilateral inferior parietal lobule has low gray matter volume in people having CPBP, compared with HCs, further suggesting that chronic pruritic disease may cause structural brain abnormalities.

### 4.5 Low gray matter volume in cerebellar regions

The posterior cerebellar lobe’s volume was significantly low in CPBP patients, according to the findings. Research into the brain center associated with chronic pruritus has suggested that the cerebellum is central to modulating chronic pruritus. For instance, patients with chronic urticaria and psoriasis exhibit altered cerebellar structure and function (Schmaal et al., 2016; Najafi et al., 2020; Wang et al., 2021). Although the cerebellum has traditionally been considered as a motor control and coordination center (Apps and Garwicz, 2005), its role in emotional regulation and cognition has been recognized (Shi et al., 2016). Furthermore, chronic pruritus induced alterations in the cerebellar gray matter volume. While existing research elucidates the cerebellum’s role in chronic pruritus-related conditions, the precise mechanism remains elusive. Hence, further investigation is warranted to delineate the cerebellum’s role in CPBP.

### 4.6 Limitations

This research has certain drawbacks. First, its cross-sectional approach made it difficult to prove a link between pruritus and microstructural alterations in gray matter, which makes longitudinal research necessary. Second, although patients with a pain VAS score > 2 were excluded, mild pain may still have affected the brain structure. Considering the high rate of comorbid pain in patients with CPBP, future studies should incorporate a control group to further exclude the influence of pain on brain morphology.

## 5 Conclusion

The temporal lobe, occipital lobe, parietal lobe, cerebellum, and frontal lobe were among the brain regions where people having CPBP showed significant decreases in gray matter volume. These structural alterations may be associated with itch-related emotions and cognition. This study investigates the CNS mechanisms underlying CPBP via imaging-based morphology, providing insights into pruritus management by targeting brain regions.

## Data Availability

The original contributions presented in this study are included in this article/supplementary material, further inquiries can be directed to the corresponding author.

## Footnotes

1
https://neuro-jena.github.io/cat/
